# Genetic diversity of Ethiopian durum wheat landraces

**DOI:** 10.1371/journal.pone.0247016

**Published:** 2021-02-17

**Authors:** Kefyalew Negisho, Surafel Shibru, Klaus Pillen, Frank Ordon, Gwendolin Wehner

**Affiliations:** 1 Ethiopian Institute of Agricultural Research (EIAR), National Agricultural Biotechnology Research Center, Holeta, Ethiopia; 2 Ethiopian Institute of Agricultural Research (EIAR), Melkassa Research Center, Melkassa, Ethiopia; 3 Martin-Luther-University, Institute of Agricultural and Nutritional Sciences, Halle (Saale), Germany; 4 Julius Kühn Institute (JKI), Institute for Resistance Research and Stress Tolerance, Quedlinburg, Germany; National Institute of Plant Genome Research, INDIA

## Abstract

Genetic diversity and population structure assessment in crops is essential for marker trait association, marker assisted breeding and crop germplasm conservation. We analyzed a set of 285 durum wheat accessions comprising 215 Ethiopian durum wheat landraces, 10 released durum wheat varieties, 10 advanced durum wheat lines from Ethiopia, and 50 durum wheat lines from CIMMYT. We investigated the genetic diversity and population structure for the complete panel as well as for the 215 landraces, separately based on 11,919 SNP markers with known physical positions. The whole panel was clustered into two populations representing on the one hand mainly the landraces, and on the other hand mainly released, advanced and CIMMYT lines. Further population structure analysis of the landraces uncovered 4 subgroups emphasizing the high degree of genetic diversity within Ethiopian durum landraces. Population structure based AMOVA for both sets unveiled significant (P < 0.001) variation between populations and within populations. Total variation within population accessions (81%, 76%) was higher than total variation between populations (19%, 24%) for both sets. Population structure analysis based genetic differentiation (FST) and gene flow (Nm) for the whole set and the Ethiopian landraces were 0.19 and 0.24, 1.04, and 0.81, respectively indicating high genetic differentiation and limited gene flow. Diversity indices verify that the landrace panel was more diverse with (I = 0.7, He = 0.46, uHe = 0.46) than the advanced lines (I = 0.6, He = 0.42, uHe = 0.42). Similarly, differences within the landrace clusters were observed. In summary a high genetic diversity within Ethiopian durum wheat landraces was detected, which may be a target for national and international wheat improvement programs to exploit valuable traits for biotic and abiotic stresses.

## Introduction

Durum wheat [*Triticum turgidum* ssp. *durum* (Desf.) Husn.] was domesticated from wild emmer (*Triticum turgidum* ssp. *dicoccoides*) to emmer (*Triticum turgidum* ssp. d*icoccum*) followed by a secondary domestication, i.e. from emmer to naked forms and durum wheat (*Triticum turgidum* ssp. *durum)* [1]. The allotetraploidization event was involved after a cross between the two diploid species: *T*. *urartu* (genome AA) [2, 3] and an unknown close relative of *Aegilops speltoides* (genome BB) [4, 5]. Thus, durum wheat has an allotetraploid genome (AABB genome, 2*n* = 4*x* = 28, seven homoeologous groups—13,000 M bp) [6]. A high-density gene-associated SNP array was developed for the characterization of polyploid wheat [7] and complemented with fully annotated high-confident genes [8]. Maccaferri et al. [9] developed the high-density tetraploid wheat consensus map from data sets of durum wheat cultivars (*Triticum turgidum* ssp. *durum*), cultivated emmer (*T*. *turgidum* ssp. *dicoccum*) and their ancestor (wild emmer, *T*. *turgidum* ssp. *dicoccoides*). Recently, the reference sequence of the genome of cv. Svevo led to the identification of 66,559 high confidence (HC) genes enabling genome-wide genetic diversity analyses in tetraploid durum wheat [10].

Durum wheat is one of the ten most important crops worldwide with an annual production of 37 million tons [11] and Ethiopia is the major durum wheat producer in sub-Saharan Africa (SSA), with a durum acreage of 0.6 million ha [11–13]. Durum wheat is primarily used for pasta production, but in addition it is used to make flour for leavened biscuits, cookies, biofuel, and for fermentation to make alcoholic beverages such as beer and liquors [14]. In the country, durum wheat nearly accounts for 15–20% of wheat production and 30% of the whole acreage [15, 16]. Hence, it contributes about 18 to 20% to the national wheat production [17, 18]. In Ethiopia, wheat (both bread and durum) is produced by around 4.62 million households with an estimated land area of 1.7 million ha and mean national yield of 2.7 t/ha [19]. Traditionally, in Ethiopia wheat straw is used as animal feed and as roof thatching material. This makes wheat biomass highly valuable in rural communities. Thus, on top of high grain yield and environmental tolerance, in wheat growing areas farmers also take into account those traits when selecting landraces.

The Ethiopian Biodivesity Institute (EBI) hosts more than 7000 landraces collected from durum wheat growers for genetic conservation and for the exploitation of genetic diversity [20, 21]. Based on the genetic diversity analysis, Mengistu et al. [22] reported a high genetic variability in Ethiopian durum wheat landraces. Kabbaj et al. [11] have demonstrated that Ethiopian durum wheat landraces cluster separatly from durum of the International Center for Agricultural Research in the Dry Areas (ICARDA), Centro Internacional de Mejoramiento de Maíz y Trigo (CIMMYT), and durum wheat derived from other countries. Genetic diversity can be described as the degree of differentiation between or within species. Existing intra-and inter-specific differences are the base of all crop improvement programs [23]. Hence, genetic variation is an essential source of novel and useful alleles to be selected by breeders for abiotic and biotic resistance/tolerance [24, 25]. It is supposed that allelic variation of genes originally found in wild species, is gradually lost through domestication and breeding [26]. Therefore, the narrowed or lost allelic variation can be recovered by exploring e.g. landraces [26]. Landraces are genetically dynamic and are in equilibrium with biotic and abiotic stresses in the environments where they evolved [27, 28]. Therefore, landraces which have adapted to their natural environment over time [29–31] can contribute favorable genomic regions for tolerance against abiotic stresses like drought.

Analysis of genetic diversity in populations is an important topic in breeding as well as conservation and evolutionary genetics studies [32, 33]. Expected heterozygosity (He) or the genetic diversity index, which is derived from gene frequency data, is used to determine the genetic variation within populations [34, 35]. Wright [36], used the fixation index (FST) to estimate genetic differentiation among populations. Leinonen et al. [37] reported that FST estimated from DNA markers provide a starting point to assess the strength of divergent selection on quantitative traits. Gene flow (Nm), which is estimated through FST is used to estimate the gene exchange within population and among populations [38]. Additionally, genetic diversity indices provide useful information on genetic diversity. Genetic analyses, such as estimation of genetic diversity and population structure, as well as genome wide association studies and marker assisted selection procedures are broadly undertaken by molecular markers [39]. Single nucleotide polymorphisms (SNPs) and simple sequence repeats (SSRs) are the most common molecular markers in genetic studies [40, 41]. Out of these, SNP markers provide an increasing resolution due to their high abundance [25, 42, 43]. Additionally, the power of SNP markers in wheat recently elevated 100-fold from 9 K [44] to 820 K [45]. In this study, we used a hybridization array that includes about 90K SNPs, which was developed for genetic analyses in allohexaploid and allotetraploid wheat populations [7, 10].

Up to now only a small part of the huge collection of durum wheat landraces hosted at EBI was characterized using SSR [46–48] and SNP markers [22, 49]. Therefore, the aim of our study was to assess population structure and genetic diversity of 215 Ethiopia durum wheat landraces, 10 released durum wheat varieties, 10 advanced durum wheat lines from Ethiopia, and 50 CIMMYT durum wheat lines using highly informative SNP markers.

## Material and methods

### Plant material

**Fig 1:**
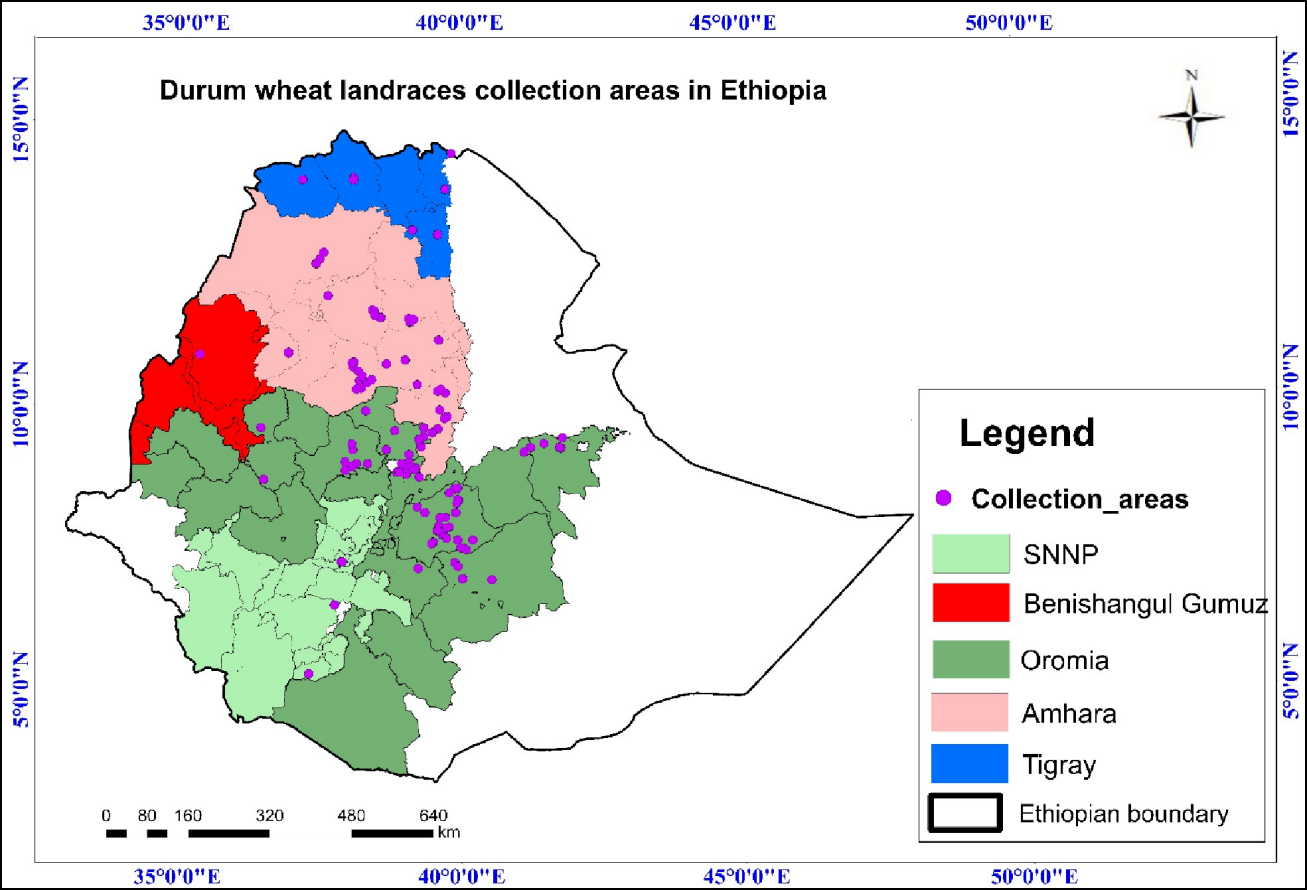
Durum wheat landraces collection areas in Ethiopia. GPS: Geographic position system, Regions of seed origin: South Nation Nationalities and Peoples (SNNP, Light green), Benishangul Gumuz (red), Oromia (green), Amhara (pink), Tigray (blue), Ethiopian boundary and geo-positions were indicated. The mapping was performed using the online ArcGIS software suite vs. 10.8.1.

A total of 285 durum wheat accessions, hereafter designated as study panel (SP) was used for the analysis of genetic diversity. The SP included, 215 Ethiopian durum wheat landraces assigned as ETDWL, 10 released durum wheat varieties, 10 advanced durum wheat lines from Ethiopia and 50 durum wheat lines from CIMMYT (S1 Table). The ETDWL were obtained from the Ethiopian Biodiversity Institute (EBI, http://www.ebi.gov.et/). Landraces were selected based on the acreage in each seed source region (origin). Thus, more samples were taken from major growing regions (Oromia and Amhara) and some samples from minor growing regions. 105 ETDWL were sampled from Oromia, 88 from Amhara, 1 from Benishangul Gumuz, 16 from Tigray and 5 from South Nation Nationalities and Peoples (SNNP), representing different seed sources (origin), seed collection zones and geographic regions (S1 Table). Online ArcGIS software was used to map the landraces collection areas in Ethiopia, https://www.arcgis.com/home/webmap/viewer.html, released version 10.8.1 July 2020 (Fig 1). For the Ethiopian durum wheat landraces, GPS passport data were obtained from EBI and are provided in S2 Table. A self-created layer was used to map positional data.

### SNP genotyping

The durum wheat SP was grown in the greenhouse at Quedlinburg, Germany for 15 days under standard growing conditions, i.e. 20 to 22°C during day time and 17 to 19°C at night [50] with automatic water supply. Genomic DNA was extracted from single plant fresh leaves following the mini-prep DNA extraction protocol [51]. Genomic DNA quality was checked by 1% gel electrophoresis and DNA concentration measurement was conducted by NanoDrop^®^ ND-1000 Spectrophotometer (Saveen Warner, Sweden). 50 ng of DNA per sample was used for SNP analysis using the 90K iSelect chip (Illumina Inc., San Diego, USA). Genotyping was conducted by Trait Genetics, Gatersleben (Germany). SNPs with a low minor allele frequency (MAF) are generally considered as rare with less power in detecting marker trait associations (MTAs) and are prone to genotyping error [52]. Thus, SNPs with minor allele frequency (MAF) of < 5%, missing data > 10% and heterozygosity > 12.5% were excluded from further analyses. Additionally, imputation was conducted using the software Beagle [53]. Physical SNP positions were taken from the reference sequence of durum wheat [8] to construct a hapmap file for further analyses.

### Population structure and genetic diversity analyses

Genotypic data were used to describe the genetic diversity within the durum wheat study panel. We analyzed the population structure and genetic diversity of the ETDWL separately, and compared this with the population structure and genetic diversity of the SP. The underlying genetic population structure was estimated with STRUCTURE 2.3.4 software [54]. SNP markers having high polymorphic information content (PIC ≥ 0.35) were selected across all durum wheat chromosomes (S3 Table). Hence, 10,000 burn-in periods followed by 100,000 Markov Chain Monte Carlo (MCMC) iterations for K = 1 to 10 clusters were used to identify the optimal range of K with five replications per cluster for the SP as well as for ETDWL. The optimal K-value was determined using the ΔK method [55]. DARwin 6.0.17 [56] was used for molecular diversity analysis to get information on genetic dissimilarity among populations and within populations. Neighbor-Joining (NJ) algorithm of the genetic distances was determined according to Saitou and Nei [57] and used to create a phylogenetic tree.

### Analysis of molecular variance (AMOVA) and genetic diversity indices

Genetic distance between populations was determined using Nei’s Genetic Distance [58] based on the number of populations k. We run AMOVA, which allowed hierarchical partitioning of genetic diversity among populations and within populations [59]. Thus, AMOVA was performed using GeneAlEx 6.503 [35]. Additionally, the genetic differentiation (FST), which is defined as a standardized measure of the genetic variance among populations was calculated to provide a measure of total genetic divergence between populations [38]. Gene flow (Nm) among populations was calculated based on FST as:

$$Nm=[(\frac{1}{{\text{F}}_{\text{ST}}})-1]/4$$

In addition, Shannon’s Information Index (I) [60], expected heterozygosity (He), unbiased heterozygosity (uHe), and the percentage of polymorphic loci (PPL) were calculated as follows:

I=−1*∑[Pi*Ln(Pi)],

He=1−∑Pi^2,uHe=[NN−1]*He.

Where Pi is the frequency of its alleles for the population and ∑*Pi*^2 is the sum of squared population allele frequencies and, *PPL* = ∑*Pi*/*N*, where Pi is the proportion of loci polymorphic in a population and N the number of populations.

## Results

### SNP analyses

**Fig 2:**
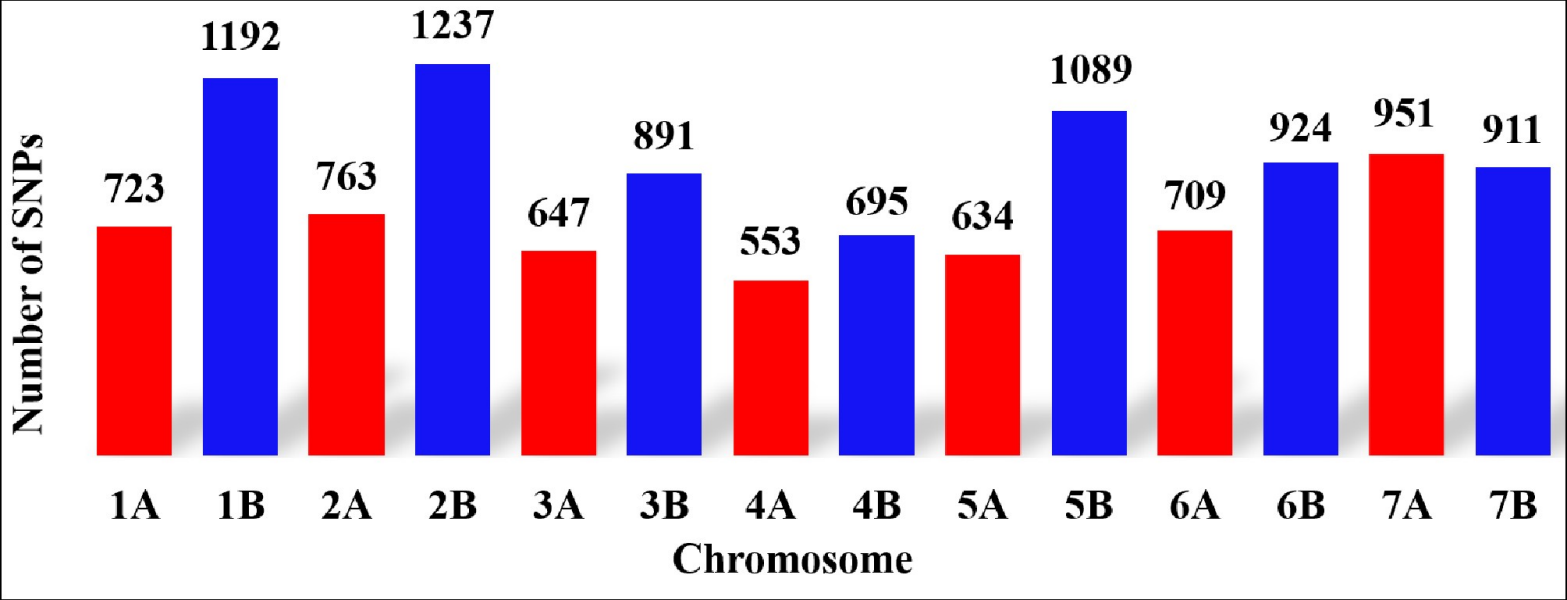
Distribution of 11,919 filtered Single nucleotide polymorphisms (SNPs) across the durum wheat genome. Genome A and B are marked with red and blue colors, respectively.

After filtering, 11,919 SNPs were used for genetic analysis. These were continuously distributed across the A and B genome of durum wheat for the SP (Fig 2). In all cases the B genome showed a higher number of SNPs except for chromosome 7, for which 951 SNPs were detected on chromosome 7A and 911SNPs on chromosome 7B. The lowest number of SNPs were detected on chromosome 4A (553) and the highest SNP number was obtained for chromosome 2B (1237). Generally, in the current study, 58% of the SNPs were located on the B genome and 42% on the A genome.

### Population structure

**Fig 3:**
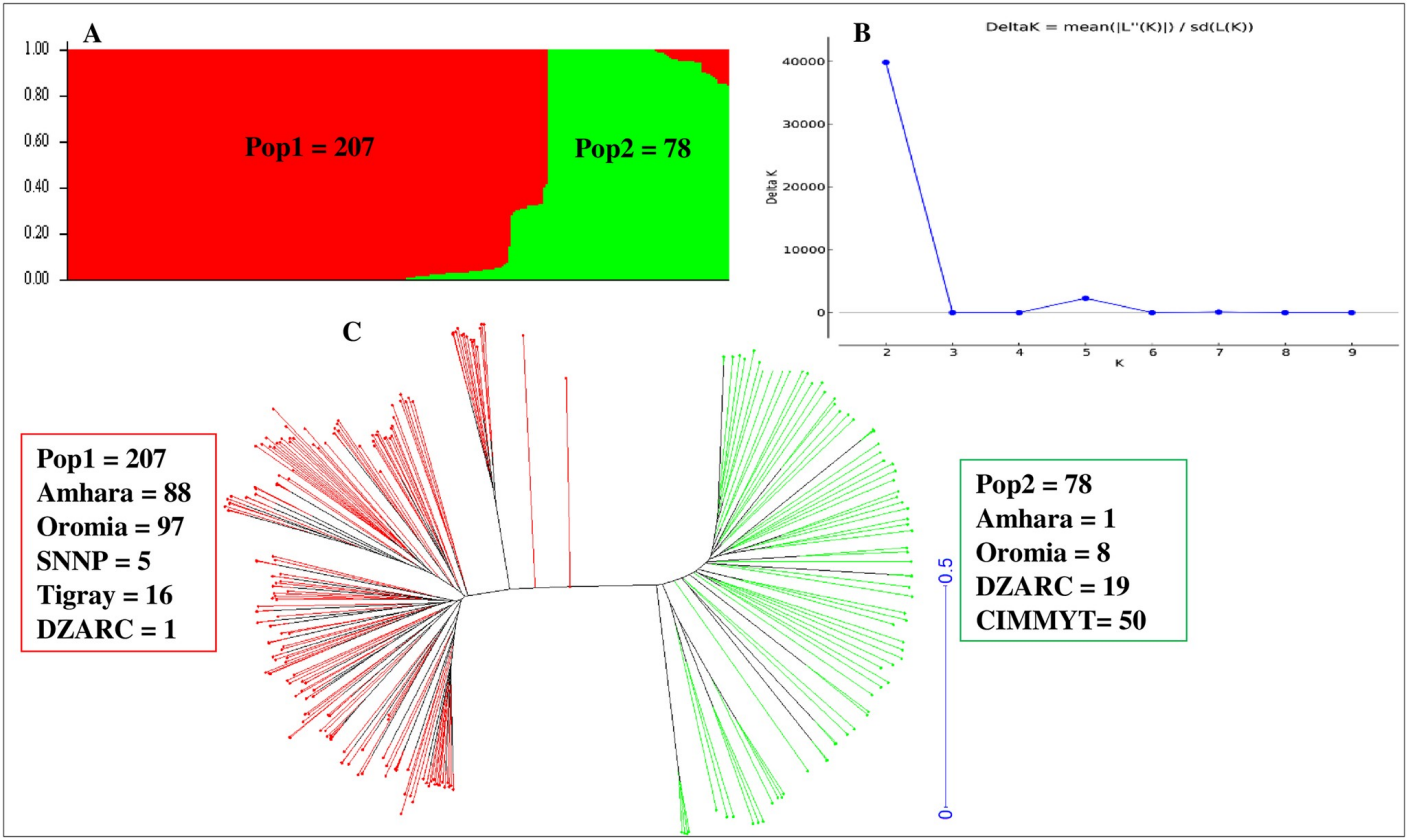
Population structure analysis of the durum wheat SP. (A) Bayesian structure analysis, (B) Structure harvester Evanno’s test ΔK at K = 2. (C) Neighbor Joining (NJ). Populations identified in STRUCTURE based on Bayesian structure analysis are shown in red and green for Pop1 and Pop2, respectively.

Population structure analysis for the durum wheat SP revealed ΔK at K = 2 i.e two populations hereafter considered as Pop1 and Pop2 (Fig 3A–3C, S1 Table). Pop1 comprised 207 accessions. Of these, 206 accessions were from ETDWL and 1 from the durum wheat lines of Ethiopia. In relation to seed origin, the number of accessions in Pop1 were originated from Amhara (88), Oromia (97), SNNP(5), Tigray(16), and Debre Zeit Agricultural research Center (DZAR, 1) (Fig 3C). Pop2 constituted of 78 accessions. Fifty of the accessions in Pop2 were from CIMMYT, 19 from the group of the released and advanced durum wheat lines of Ethiopia and 9 were landraces. The landraces clustered in Pop2 were DW006, DW007, DW008, DW020, DW039, DW143, DW185 and DW188 from Oromia, as well as DW050 from Amhara (Fig 3C, S1 Table). Thus, the SP mainly split in the ETDWL and advanced varieties.

**Fig 4:**
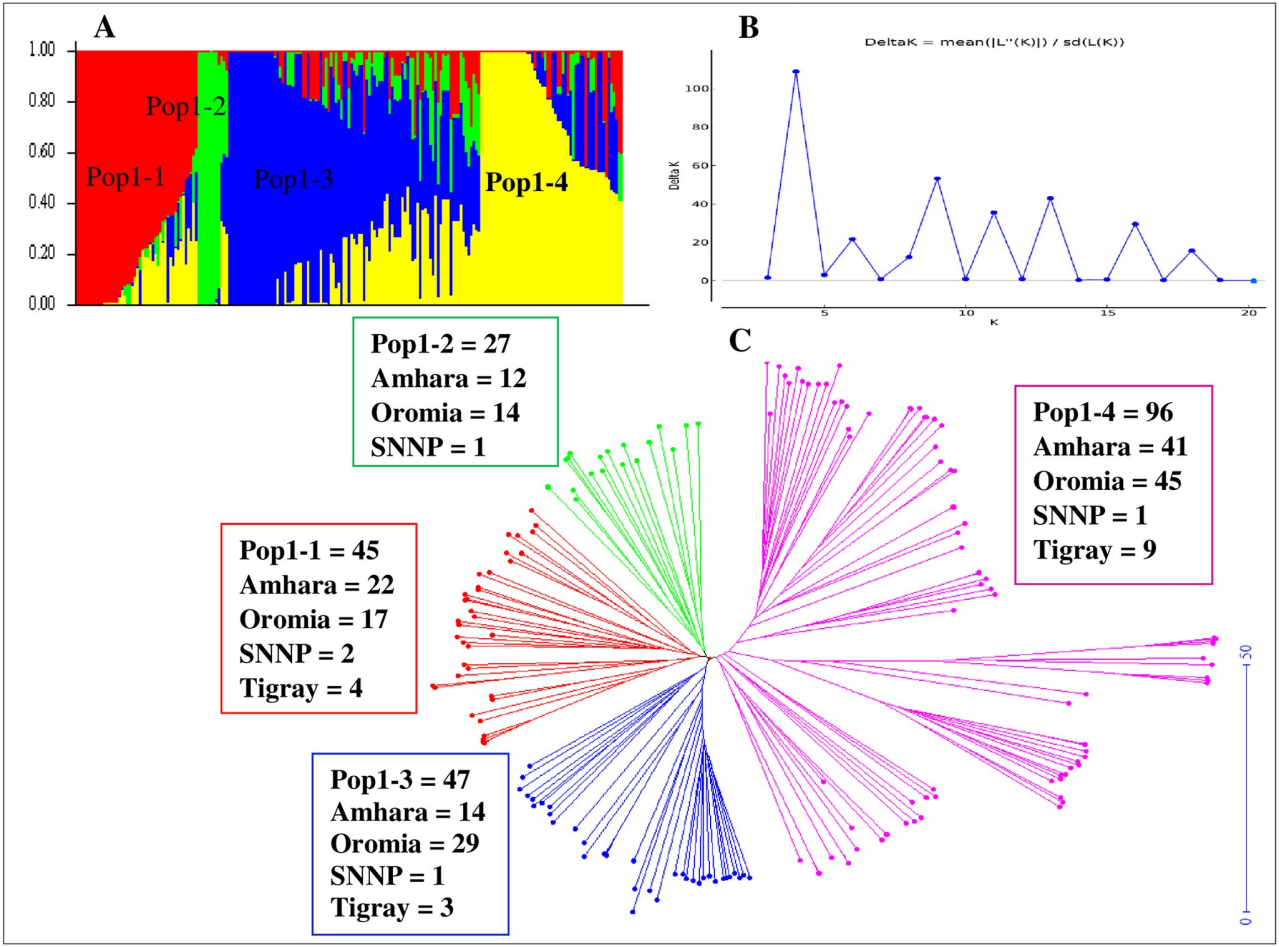
Population structure analysis of ETDWL. (A) Bayesian structure analysis, (B) Structure harvester ΔK at K = 4. (C) Neighbor-Joining (NJ). Populations identified in STRUCTURE based on Bayesian structure analysis are shown in red, green, blue and yellow/pink, for Pop1-1 to Pop1-4, respectively.

Ethiopian durum wheat landraces (ETDWL) comprise accessions collected from major wheat producing regions of the country (Oromia, Amhara, Tigray and SNNP) (Fig 1, S1 Table). Population structure analysis of the ETDWL uncovers populations with ΔK at K = 4 (Fig 4A–4C, S1 Table). The populations in ETDWL comprised 45, 27, 47, and 96 accessions, respectively for Pop1-1, Pop1-2, Pop1-3, and Pop1-4 (Fig 4C). Pop1-1 comprises 45 accessions of which 22 originated from Amhara, 17 from Oromia, 2 from SNNP and 4 from Tigray. In the second cluster (Pop1-2) which comprises 27 accessions,12 accessions were from Amhara, 14 from Oromia and 1 from SNNP. Pop1-3 consisted of 47 accessions, i.e. 14 from Amhara, 29 from Oromia, 1 from SNNP and 3 from Tigray. Pop1-4 comprises 96 accessions of which 41 derived from Amhara, 45 from Oromia, 1 from SNNP and 9 from Tigray. The number of accessions per cluster in ETDWL ranged from a minimum of 27 to a maximum of 96 for Pop1-2 and Pop1-4, respectively.

### Analysis of molecular variance (AMOVA)

Analysis of molecular variance (AMOVA) for the durum wheat SP and ETDWL was conducted taking the respective population structure clusters (ΔK) into account (Table 1). In both cases, AMOVA indicated significant (P < 0.001) effects for variation between populations and within populations. The AMOVA of the SP revealed that 19% of the total variation is between populations, while 81% of the total variation is present within populations. Fixation index (FST) and gene flow (Nm) for the SP were calculated at FST = 0.19 and Nm = 1.04, respectively. Similarly, AMOVA for ETDWL revealed 24% of the total variation between populations and 76% variation within populations. Fixation index (FST) and gene flow (Nm) for the ETDWL were FST = 0.24 and Nm = 0.81, respectively. Therefore, the AMOVA for SP and ETDWL showed higher percentage of variation within populations than between populations (Table 1).

**Table 1:** AMOVA for the SP and ETDWL based on structure analysis results.

Source of variation	df	Sum of squares	Variance components	Percentage of variation	Fixation index (Fst)	Gene flow (Nm)
**Variance partition of the SP, k = 2**						
**Between populations**	1	5323.9***	22.7	19	0.19	1.04
**Within populations**	283	53223.7***	94.0	81		
**Variance partition of the ETDWL, k = 4**						
**Between Populations**	3	7069.6***	23.0	24	0.24	0.81
**Within populations**	211	31572.1***	74.8	76		

df: degree of freedom,

***: P-value at P < 0.001,

SP: Study panel, ETDWL: Ethiopian durum wheat landraces.

### Genetic indices

We investigated the genetic diversity of the SP and ETDWL based on population structure analysis results of ΔK at k = 2 and k = 4, respectively (Table 2). The genetic indices for the SP such as I, He, and uHe showed higher values for Pop1 as compared to Pop2. Hence, Pop1 that comprised 99.5% accessions from ETDWL was more diverse (I = 0.7, He = 0.46, uHe = 0.46) than Pop2 (I = 0.6, He = 0.42, uHe = 0.42), which comprised 88.5% of improved varieties (advanced, released and CIMMYT durum wheat). Pop1-3 of the ETDWL was the most diverse (I = 0.62, He = 0.39, uHe = 0.39) with 100% PPL followed by Pop1-2 (I = 0.52, He = 0.33, uHe = 0.34) with 89.8% PPL. Pop1-1 and Pop1-4 showed similar genetic diversities (I = 0.5, He = 0.32, uHe = 0.32) with 93.1% and 97.6% PPL, respectively (Table 2).

**Table 2:** Mean of different genetic indices parameters in each population.

Pop	N	I	He	uHe	PPL
**Population of the SP, K = 2**					
**Pop1**	207	0.7	0.46	0.46	100
**Pop2**	78	0.61	0.42	0.42	100
**Population of the ETDWL, k = 4**					
**Pop1-1**	45	0.5	0.32	0.32	93.1
**Pop1-2**	27	0.52	0.33	0.34	89.8
**Pop1-3**	46	0.62	0.39	0.39	100
**Pop1-4**	96	0.5	0.32	0.32	97.6

Number of accessions (N), Shannon’s information index (I): *I* = −1 * ∑[*Pi* * *LnPi*], expected heterozygosity (He) or genetic diversity: *He* = 1 − ∑*Pi*^2, Unbiased heterozygosity $uHe=[\frac{N}{N-1}]*He$, and percentage of polymorphic loci: *PPL* = ∑*Pi*/*N*; SP: Study panel, ETDWL: Ethiopian durum wheat landraces.

## Discussion

Hybridization arrays are believed to represent a significant fraction of SNPs distributed across genomes. In wheat they represent SNPs between populations of diverse geographical origin [7, 44, 45]. Hence, in this study, we used the hybridization array that includes about 90K SNPs, which was developed to analyze genetic variation in allohexaploid and allotetraploid wheat populations [7, 10]. Studies indicated a higher number of SNPs in the B than in the A genome of wheat [61, 62]. Likewise, higher number of SNPs was also identified in this study on the B genome (58%) than on the A genome (42%) (Fig 2). However, we detected a higher number of SNPs on chromosome 7A (951) than on chromosome 7B (911). Similarly, studies by Naz et al. [63] and Desta et al. [64] on bread wheat indicated highest numbers of SNP markers on the B genome followed by the A genome and less across the D genome.

In this study, population structure and Neighbor-Joining (NJ) analysis showed two populations (Pop1 and Pop2) for the study panel (SP). Concerning Pop1, 206 (99.5%) accessions were from ETDWL and only 1, DZ005 (0.5%) from the advance durum wheat lines (Fig 3). This durum wheat line most probably was selected from landraces by Ethiopian durum wheat breeders. This elucidates that only little effort was spent to include landraces in durum wheat improvement programs in the country. Pop2 of the SP constituted of 69 (88.5%) accessions from CIMMYT and others that originate from international sources like ICARDA which are released durum wheat varieties and advanced durum wheat lines. The remaining 9 (11.5%) accessions are landraces. The landraces clustered in Pop2 were most probably incorrect renamings of the released durum wheat varieties as landraces during germplasm collection or they may be an admixture. In Ethiopian since 1970 until recently, CIMMYT is the major source for most of the improved durum wheat materials [23]. In support of this, this study clearly showed that 19 out of 20 Ethiopian accessions plus advanced durum lines are clustered in Pop2 with durum wheat lines from CIMMYT. This is possible under the scenario that most improved durum wheat materials were introduced from international breeding programs to the country [13]. Additionally, it shows that only little attention was given to explore the genetic diversity in ETDWL as pointed out by [22]. Therefore, in Ethiopia to exploit the existing genetic diversity more focus should be given to conserve and use the landraces in durum wheat breeding programs.

It has been reported that, Ethiopian durum wheat landraces are distinct and have no kinship with the Middle-East, which is the primary region of origin of durum wheat [11]. Therefore, the separate clustering of Ethiopian durum landraces from international varieties may illustrate a long-time separation of Ethiopian durum wheat landraces from primary durum origin and from international germplasm. This is attributed to the uniqueness of Ethiopian durum wheat landraces [11, 13, 22, 46]. This is in agreement with reports that designated Ethiopian durum wheat landraces as separate sub-species under the name *T*. *durum subs*. *abyssinicum or T*. *aethiopicum* [22, 23]. Additionally, separate clustering of Ethiopian durum wheat from improved durum wheat in Ethiopia indicated that little or no improved varieties were generated from landraces either through selection or via crossing with international durum wheat materials. Nevertheless, germplasm originating from international organization such as CIMMYT and ICARDA remain the main source for advanced durum lines and released durum varieties in Ethiopia [13].

Population structure analysis of ETDWL alone uncovers four populations (Pop1-1, Pop1-2, Pop1-3 and Pop1-4), which is in agreement with and NJ analysis (Fig 4). Mengistu et al. [22] have identified a number of populations (k = 10) in Ethiopian durum wheat landraces by removing improved durum wheat varieties from the population analysis. Our study also signifies the presence of higher admixture of accessions between different populations of landraces (Fig 4). This is a common phenomenon for most cereal crops grown in Ethiopia because of informal seed exchange systems involving regional and countrywide farming communities. In Ethiopia, farmers exchange seeds of cereals in various traditional forms such as gifts, barter, labor exchange or social obligations [65, 66]. Therefore, the main source of seed for planting wheat and barley landraces in Ethiopian smallholder communities is via the informal farmer to farmer seed exchange. Apparently, once farmers obtain seed with required quality that genotype will get bigger chance to spread across local communities. This was demonstrated by genetic clustering based on seed collection regions where seeds originated from one region relatively closely clustered in the same population. For instance, 9 out of 16 accessions collected from Tigray were clustered in Pop1-4, on the other hand no accession from this region was grouped in Pop1-3 (Fig 4). The geographic isolation and latitudinal variation, i.e. 1540–3190 meter above sea level from which accessions were collected, confirmed the variability and genetic dynamics in Ethiopian durum wheat landraces to adapt to wide-ranging conditions (Fig 1, S1 Table). Subsequently, the high-level of genetic diversity can be exploited in wheat breeding and improvement programs to overcome the biotic and abiotic stresses across latitudinal ranges.

Durum wheat is one of the important cereal crops grown in Ethiopia and the country is endowed with a wealth of genetic diversity for tetraploid wheat. Phenotypic and morphological analysis [23, 67–69] and genotypic analysis elucidated the existance of huge genetic diversity in ethiopian tetraploid wheat [22, 48, 70]. Consquently, the country is considered as the center of diversity and/or secondry center of origion for durum wheat [11, 22, 71]. In our study, genetic diversity within population accessions was higher than genetic diversity between populations (Table 1) illustrating that more attention should be given to individual accessions within populations to explore the existing genetic diversity as a basis for genomic analysis, and for genetic material conservation.

Fixation index (differentiation = FST) measures population differentiation due to genetic structure [72] and FST can be considered important in differentiating populations when its value is greater than 0.15 [73]. Hence, FST values were calculated at FST = 0.19 and FST = 0.24 for the SP and ETDWL, respectively indicating significant differentiations between the populations. Eventually, in our study, the higher genetic differentiation led to limited gene flow (Nm) values of Nm = 1.04 and Nm = 0.81 for the SP and ETDWL, respectively (Table 1). Nm value less than one is an indication of limited gene exchange as it was suggested by [38, 73]. Therefore, the Nm < 1 in ETDWL (0.81) clearly shows the high degree of genetic differentiation that exists among the ETDWL populations (FST = 0.24) as compared to SP (FST = 0.19) [37, 74]. In agreement to this, [39] reported that a high genetic exchange lead to low genetic differentiation between populations. Similarly [22], reported high genetic diversity in Ethiopian durum wheat landrace collections. Apparently, in the present study, population structure analysis of ETDWL alone revealed more populations suggesting the huge genetic diversity that exists within Ethiopian durum wheat landraces (Fig 4). Information on genetic diversity of each population can be assessed using genetic diversity indices [39]. Likwise, in this study, diversity analysis was further supported by the genetic diversity indices such as I, He, and uHe (Table 2). Genetic diversity indices for the SP illustrated higher genetic diversity in Pop1, which constituted 99.5% of the ETDWL as comparison to Pop2, which comprised only 11.5% ETDWL. Genetic diversity indices for the ETDWL indicated that Pop1-3 was the most diverse followed by Pop1-2, whereas Pop1-1 and Pop1-4 showed similar genetic diversity (Table 2). This marked that landraces showed huge genetic diversities that can broaden the genetic base for wheat improvement. In agreement to this, biotic and abiotic resistance/tolerance genes or genomic regions were identified in Ethiopian durum wheat landraces, e.g. resistance to biotic factors such as stripe rust resistance [75], adult plant resistance to leaf rust and stem rust [46, 76, 77], abiotic stress resistance such as aluminum tolerance [78] and terminal drought tolerance [23]. Therefore, Ethiopian durum wheat landraces may increase the rate of genetic gain if strategically included in wheat breeding programs. Most important, exploiting the landraces genetic diversity in Ethiopian durum wheat may help to mitigate abiotic stress factors that are apparent due to adverse effects of climate change. Furthermore, these landraces may help to uncover unknown genomic regions or genes associated with economically important traits.

## Conclusion

We employed high quality SNP markers to analyze the population structure and genetic diversity of a durum wheat study panel comprising 285 accessions of which 215 accessions were ETDWL. AMOVA (P < 0.001) unveiled that genetic variation within population accessions was higher than genetic variation between populations for the SP and ETDWL. Structure analysis of SP revealed two distinct populations (Pop1 and Pop2). Genetic diversity indices for the SP illustrated higher genetic diversity in Pop1, which constituted 99.5% of the ETDWL in comparison to Pop2, which comprised only 11.5% ETDWL. Further population structure analysis of the ETDWL alone uncovered four populations emphasizing the high degree of genetic diversity that exists in ETDWL. Genetic diversity indices for the ETDWL indicated Pop1-3 was the most diverse followed by Pop1-2. Therefore, the high genetic diversity detected in ETDWL showed the existence of plentiful variability that could be utilized for future wheat breeding programs.

## Supporting information

S1 Table Population structure analysis results for SP. n = 285 with Delta K at k = 2 and ETDWL, n = 215 with Delta K at k = 4.
(XLSX)

S2 Table Passport data of the Ethiopian durum wheat landraces. (XLSX)

S3 Table Highly informative selected 420 SNP markers for genetic diversity analysis and stratification. (XLSX)
